# Cortical activation for adolescent-onset minor depression and major depressive disorder: an fNIRS study

**DOI:** 10.1186/s12991-024-00500-6

**Published:** 2024-05-09

**Authors:** Gaizhi Li, Ke Ma, Kathryn Rossbach, Ying Niu, Qiqi Li, Zhifen Liu, Kerang Zhang

**Affiliations:** 1 Department of Psychiatry, First Hospital of Shanxi Medical University, No 85 Jiefang Nan Road, Taiyuan, Shanxi Province 030001 China; 2 First Clinical Medical College, Shanxi Medical University, Taiyuan, Shanxi Province China; 3 Department of Orthodontics, First Hospital of Shanxi Medical University, Taiyuan, Shanxi Province China; 4 College of Medical Sciences, Shanxi Medical University, Taiyuan, Shanxi Province China; 5 Rossbach Psychological Services, Rosswell, GA, 30075 USA

**Keywords:** Adolescent, Minor depression, Major depressive disorder, fNIRS

## Abstract

**Background:**

While depression is increasing worldwide, some patients are diagnosed as having Major Depressive Disorder (MDD), but others are diagnosed with minor depression, however, the potential neuro mechanism is unknown.

**Methods:**

Sixty-two patients with minor depression, 44 adolescents with MDD and 54 healthy adolescents participated in this study. Functional near-infrared spectroscopy (fNIRS), both HAMD and HAMA data were collected from all of the participants.

**Results:**

The result indicates the pervasively decreased activation of BA, 11, 21, 45 and 46 were observed in the MDD group and reduced activation of BA 45 was observed in the minor depression group. However, cortical activation was not observed between the minor depression or MDD groups. Cortical activation was also not correlated with the depressive/anxious score in the minor and MDD groups separately.

**Conclusions:**

Cortical activation was pervasively decreased in the MDD group and slightly reduced in the minor depression group, which may be a potential neural mechanism. As reduced cortical activation in minor depression, interventions in the early stages of minor depression may help slow or even modify the development of the illness.

## Introduction

Depression is characterized by depressed mood, lack of interest and low energy, with additional clinical symptoms including poor concentration, feelings of guilt or worthlessness, appetite changes, sleep disturbances, psychomotor retardation or agitation, suicide attempt or thoughts, et al. There are several different subtypes of depression based on specific clinical symptoms (melancholic depression, psychotic depression), onset (seasonal affective disorder, postpartum, early versus late in life), course (single, recurrent, chronic), and severity. According to Diagnostic and Statistical Manual of Mental Disorders (DSM-5), depression have severity specifiers as mild, moderate and severe, also other specifiers such as with anxious distress, mixed feature, et al. When looking at patients suffering from depressive symptoms, some of them meet the criteria of full-blown Major Depressive Disorder (MDD), however, others do not meet the diagnostic criteria (such as clinical symptoms, severity, or illness duration), who are often regarded as having minor (or subsyndromal/subthreshold) depression. Minor depression was defined as ‘having two or more depressive symptoms for at least two weeks, accompanied by social dysfunction’ by Judd, et al. [1].

With regard to the treatment of antidepressants, patient-level meta-analysis showed minimal or non-benefit for patients suffering from minor depression and substantial benefit for patients having MDD [2].

The prevalence of minor depression ranges from 2.5 to 16% in community and primary care settings, and the prevalence is particularly higher in elderly patients [3, 4]. Individuals with minor depression are at higher risk of developing MDD and are more likely to have depressive episodes [5], as 12% of individuals with minor depression developed into MDD after a 3-year follow-up [6]. Risk factors for developing MDD from minor depression include poor social support, recurring short episodes of depressive symptoms, anxiety disorders, substance use disorders, suicidal thoughts across the lifetime, a chronic physical problem, and diminished social functioning, et al. Though minor depression occurs at a higher prevalence and causes a greater burden to personal, family and society, less is known about the diagnosis of minor depression.

Previous studies about neural correlates of minor depression focused on late-life patients. Fewer studies paid attention to adolescents. However, adolescents with depression are increasing, with a large number of adolescents suffering from minor depression [7]. Ghazi et al. used DTI (Diffusion Tensor Imaging) to explore the mechanism of young adults with subthreshold depressive symptoms and observed significantly reduced microstructural changes (using fractional anisotropy value, FA) in the depression symptoms group compared to the control group [8]. Polyakova et al. did not observe the difference in cortical thickness between the minor depression group and healthy subjects [9], which is also not observed in late-life minor depression and health controls [9].

fMRI was used by previous studies to investigate biomarkers of MDD and minor depression. However, most studies explored the difference between MDD and HC, and pervasively differentiated brain areas were observed between the MDD and HC groups, such as DLPFC, precuneus, amygdala, et al. [10–12]. The underlying neural mechanism for depressive symptoms was reported, however still lacking, for example, the changes in the left hippocampus and left caudate nucleus nodal centrality was correlated with the severity of depression severity (HAMD scores) [12]; some studies focused on minor depression and HC [13, 14], as left middle frontal gyrus(MFG), right precuneus, superior frontal gyrus(SFG), and hippocampus were reported; a significant positive correlation between Beck Depression Inventory- II (BDI-II, a 21 items self scale for assessing the severity of depression, higher score indicating severe depressive symptoms) scores and resting-state brain functional connectivity from MFG to hippocampus was reported. Fewer studies investigated the difference of MDD and minor depression. These studies suggest that MDD and minor depression lead to changes in a wide range of brain regions. The high spatial resolution of fMRI provided advantage for research, but the price limited its use. Functional near-infrared spectroscopy (fNIRS) has higher temporal resolution, lower cost, is harmless, and provides unlimited application scenes, which has been widely used for understanding mental disorders, including schizophrenia, bipolar disorder, depressive disorder, and other mental illness [15, 16]. fNIRS is used to measure the changes of oxygenated hemoglobin and deoxygenated hemoglobin at the cortical level (frontal and temporal areas) [17–21].

Previous reports compared the neuro mechanism of depression and healthy controls using fNIRS, for example, Dong et al. [22] observed reduced prefrontal activation (bilateral VLPFC and OFC) in the MDD group. Kim et al. [23] compared the change of oxy-Hb in young adults (18–34 years old) with MDD and suicidality. They found prefrontal oxygenation (right VLPFC) was lowest in MDD group with suicidality, compared with MDD group and healthy controls. Wang et al. [24] divided 41 college students into higher and lower depressive tendencies groups based on their BDI scores. Higher depression groups showed higher deactivation of oxyhemoglobin (HbO) in the superior external frontal cortex (BA46), inferior frontal gyrus (BA45), premotor cortex (BA6), and primary motor cortex (BA4). Liu et al. reported that significantly lower cortical activation was demonstrated in the prefrontal cortex (PFC) in adolescents with depression [25].

The current study hypothesizes that frontal and temporal cortex activation is lower in the minor depression group compared with the HC group, and higher compared with the MDD group. The present study also aimed to examine the neurological mechanisms of clinical symptoms (depressive and anxious symptoms) using fNIRS in MDD and minor depression groups separately.

## Methods

### Participants

Forty-four adolescents with MDD were recruited for the current study between January and December 2021. Recruitment was based on patients’ admission to the Children and Adolescents Outpatient Department of Psychiatry and Mental Health, the First Hospital of Shanxi Medical University. The inclusion criteria consisted of: (1) aged 12 to 23 years old; (2) diagnosed with DSM-5 MDD (Major depressive disorder is diagnosed with at least two weeks of persistent depressed mood, loss of interest, or hopelessness co-occurred, also with five additional symptoms affecting social, working functioning). Exclusion criteria consisted of having any other mental disorder diagnosis which was assessed by the psychiatrist of the study using the Mini-International Neuropsychiatric Interview (M.I.N.I.) [26].

Sixty-two adolescents with minor depression were also recruited from the Children and Adolescents Outpatient Department of Psychiatry and Mental Health, the First Hospital of Shanxi Medical University. The inclusion criteria consisted of: (1) aged 12 to 23 years old; (2) minor depression was assessed by the trained psychiatrist (Dr. Li and Dr. Liu) according to the diagnosis criteria suggested by Judd, et al. [1]. Exclusion criteria consisted of being diagnosed with any other mental disorder which was assessed by the psychiatrist of the study using the Mini-International Neuropsychiatric Interview (M.I.N.I.) [26].

We also recruited 54 healthy controls locally from advertisements. The inclusion for the HC including aged 12–23 years old, without gender limitation. The M.I.N.I. was used for the exclusion of other mental disorders.

### General demographic data

Demographic data including age, gender, and number of educational years were collected from all of the participants.

### Functional near-infrared spectroscopy (fNIRS)

The hemodynamic responses in the prefrontal and superior temporal cortices were measured by a 52-channel fNIRS system with 17 transmitters and 13 receivers (ETG-4100. Hitachi Medical Co., Tokyo, Japan). The fNIRS system contains 52 measurement channels, which measure hemodynamic response in the bilateral prefrontal cortices, and the superior parts of the temporal cortex, as can be seen below. The Verbal Fluency Test (VFT) was taken concurrently with the fNIRS data collection. The VFT is widely used in fNIRS because of its ability to effectively respond to participants’ brain hemodynamic activation. Participants completed the VFT in a quiet environment. The participants sat down with their eyes open, avoided excessive head and body movements, and focused on the cross on the screen. The VFT consisted of three periods: the first period was the pre-task period (30 S), in which the participants repeated “one, two, three, four, five”; The second period was the task period (60 S), in which the participants had to name as many four-word idioms or phrases starting with the words “Big, White, Sky” as possible in the 60 S period. The correct, non-repeated idioms or phrases were recorded as the participants’ VFT scores; the third period was the post-task period (70 S), in which the participants also repeated “one, two, three, four, five” [25].

### fNIRS statistical analysis

#### fNIRS data preprocessing

Data were preprocessed using MATLAB 2013b and the NIRS-SPM toolbox. The NIRS-SPM toolbox is a MATLAB-based software package for processing and statistical analysis of near-infrared spectroscopy signals [27, 28].

(1) Converts all .csv files to NIRS-SPM available .mat files.

(2) Check for normal and available channels for all participants.

#### Calculate the value of β

The NIRS-SPM toolbox is based on general linear models (GLM). Y = βx + ε is the equation of the GLM. β is the coefficient of fit, which in this study represents the level of cortical activation induced by the VFT.

(1) Low-frequency drift due to breathing, heartbeat, vascular pulsations, or other experimental factors was removed using the discrete cosine transform (DCT). In the second step, physiological noise in the data was filtered using a low-pass filter based on the hemodynamic response function (HRF).

(2) A general linear model was built. The time series related to task rest and task performance were used as the independent variables, the oxyhemoglobin concentration as the dependent variable, and the first-order derivative and second-order derivative of the time series as the covariates.

(3) The value of β in the GLM model is calculated for every independent variable.

#### Index extraction

**Fig. 1:**
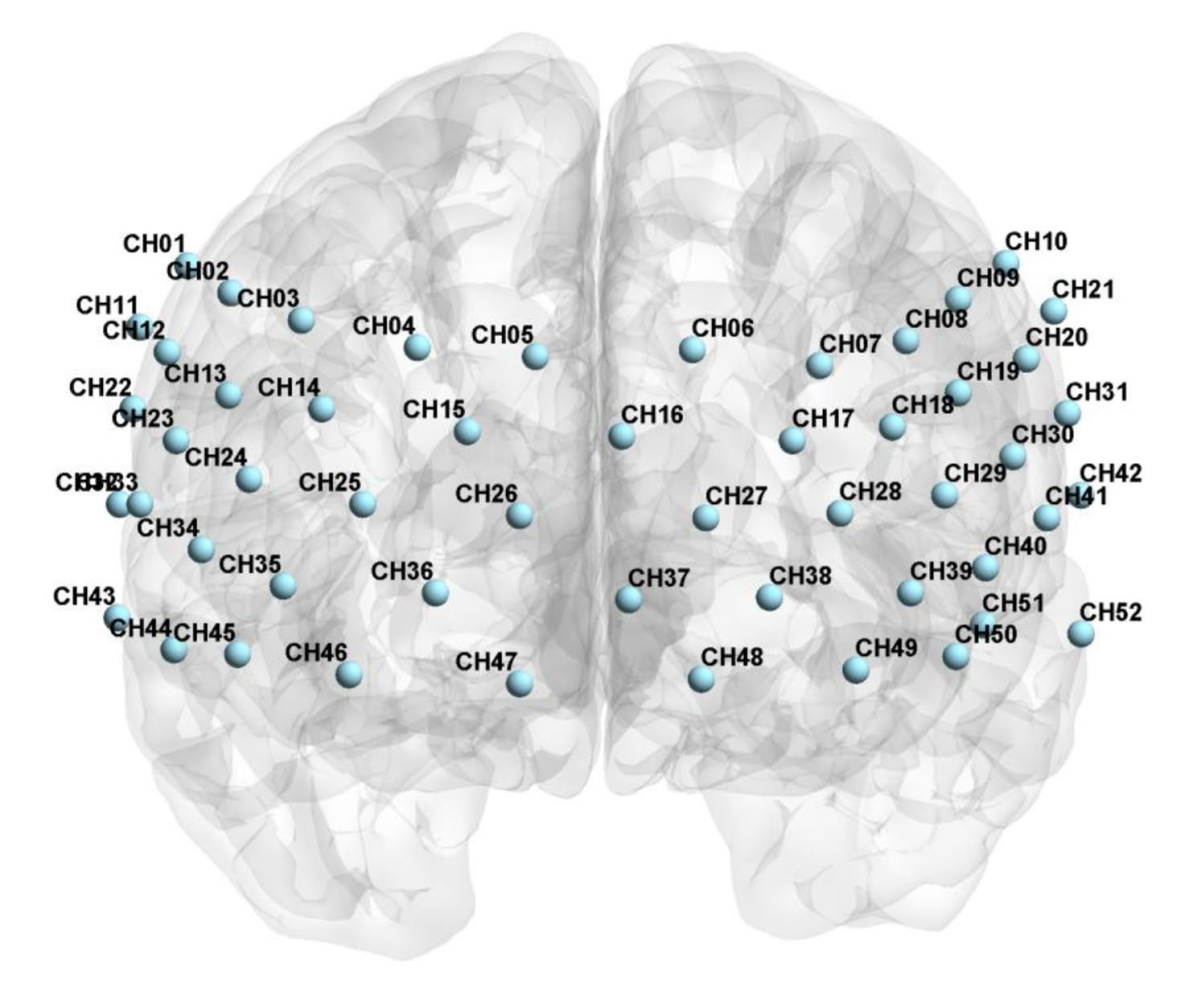
The distribution of 52 channels

(1) The values of β were extracted for all channels for every participant. The Δβ value of oxy-hemoglobin is equal to VFT β value minus baseline β value, which is used to measure the activation of the frontal and temporal cortex during the VFT task.

(2) The 52 channels (See Fig. 1) were grouped into six anatomic macro-areas for further analysis, including DLPFC, Frontopolar area, Subcentral area, OFC, MTG and Pars triangularis. The mean Δβ values of all channels within every brain region were extracted.

### Statistical analysis

The continuous data were analyzed using ANOVA (with age, gender and educational years as covariates) and categorical data were analyzed with the chi-square test. Bonferroni test was used for post-hoc analysis. The correlation of Δβ value and clinical symptoms was analyzed with age, gender and educational years as covariates. All of the tests were two- tailed, and the *p*-value was corrected by false discovery rate (FDR) [29, 30], with P(FDR) < 0.05 as significant. All the data were analyzed using SPSS 22.0 (SPSS Inc, Chicago, IL, USA).

## Results

### Demographic data and clinical characteristics

The minor depression group was the youngest group, followed by the MDD group, then the HC group. The number of years of education of the minor depression and MDD group was also lower compared with the HC group. Both the HAMD and HAMA scores of the MDD group were higher than the minor depression group, and the minor depression group was higher than the HC group. See Table 1.

**Table 1:** Demographic and clinical characteristics of the participants

	Minor depression group (*n* = 62)^a^	MDD group (*n* = 44)^b^	HC group (*n* = 54)^c^	F/χ^2^	*p*	Post-hoc
Age	15.29 ± 1.69	16.70 ± 2.71	19.22 ± 2.58	41.952	< 0.001	a < b < c
Gender	20:42	12:32	8:46	4.851	0.088	-
Years of Education	9.92 ± 1.74	10.20 ± 2.75	13.65 ± 2.92	37.984	< 0.001	a < c, b < c
HAMD	16.00 ± 3.11	24.59 ± 7.49	1.40 ± 2.27	309.056	< 0.001	c < a < b
HAMA	13.37 ± 4.09	15.34 ± 4.90	1.16 ± 2.07	197.972	< 0.001	c < a < b

### Hemodynamic response during the verbal fluency test

**Fig. 2:**
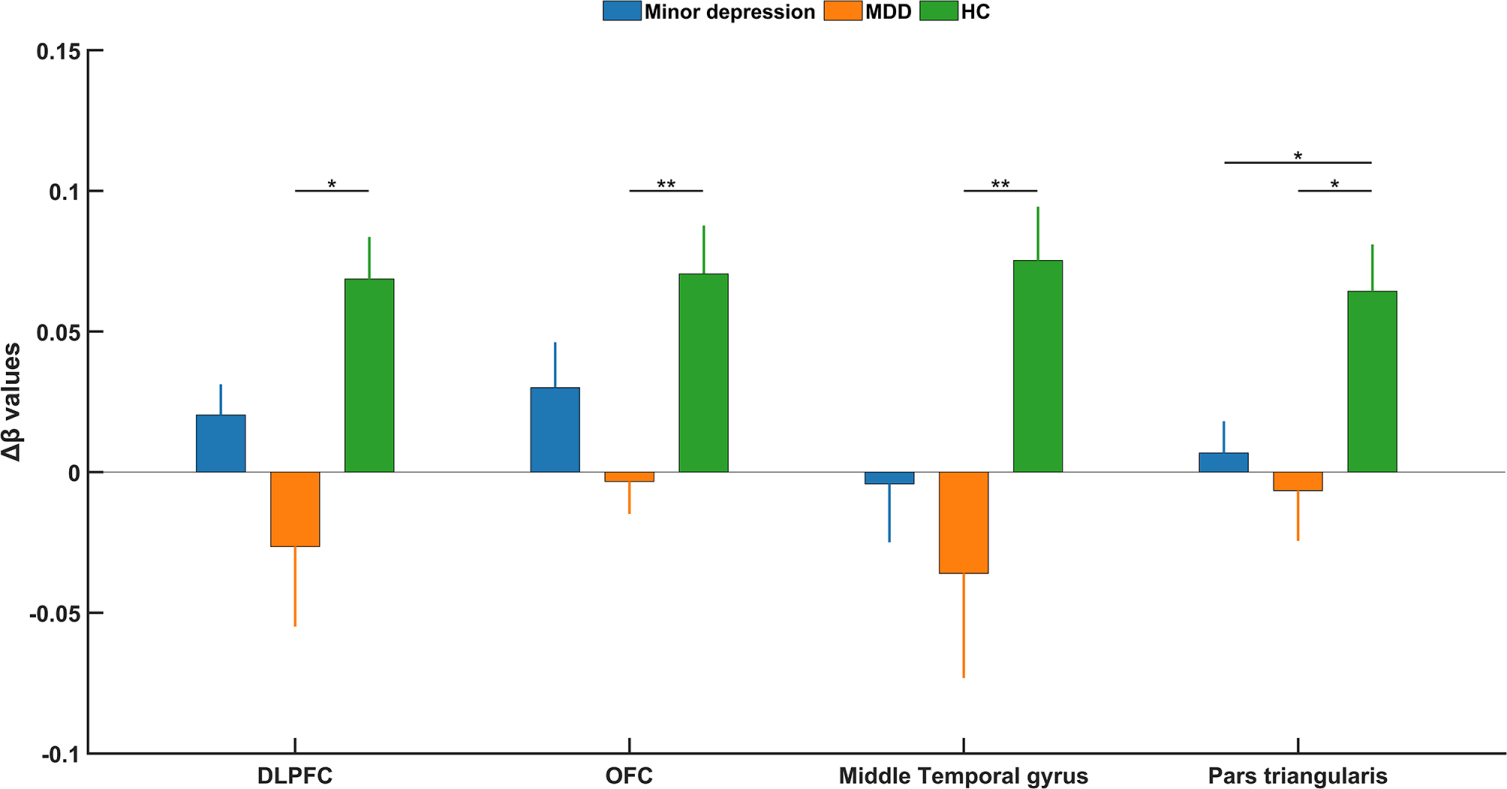
The Δβ value of brain regions among the three groups

The Δβ value of the dorsolateral prefrontal cortex (DLPFC), orbitofrontal cortex (OFC), middle temporal gyrus (MTG) and Pars triangularis in the MDD group was significantly lower compared with the HC group (*p* < 0.05), the Δβ value of Pars triangularis in the minor depression group was also lower than HC group; see Table 2; Fig. 2. The Δβ value in the Frontopolar area and Subcentral area among the three groups was not of great significance.

**Table 2:** Comparison of Δβ during VFT among the three groups

	Minor depression group (*n* = 62)^a^	MDD group (*n* = 44)^b^	HC group (*n* = 54)^c^	F	*p*	Post-hoc FDR
DLPFC (BA 46)	0.0203 ± 0.0857	-0.0264 ± 0.1889	0.0686 ± 0.1100	4.4366	0.0234	b < c
Frontopolar area (BA 10)	0.0243 ± 0.0745	-0.0692 ± 0.3750	0.0602 ± 0.1077	3.1819	0.0620	-
Subcentral area (BA 43)	-0.0944 ± 0.4235	-0.0607 ± 0.1426	0.0253 ± 0.1194	1.4236	0.2847	-
OFC (BA 11)	0.0301 ± 0.1270	-0.0033 ± 0.7679	0.0705 ± 0.1262	5.4432	0.0218	b < c
MTG (BA 21)	-0.0042 ± 0.1637	-0.3599 ± 0.2467	0.0752 ± 0.1403	4.8164	0.0218	b < c
Pars triangularis (BA 45)	0.0068 ± 0.0887	-0.0065 ± 0.1187	0.0643 ± 0.1220	5.1292	0.0218	a < c, b < c

### Correlation of clinical symptoms and Δβ values in the ROIs

**Fig. 3:**
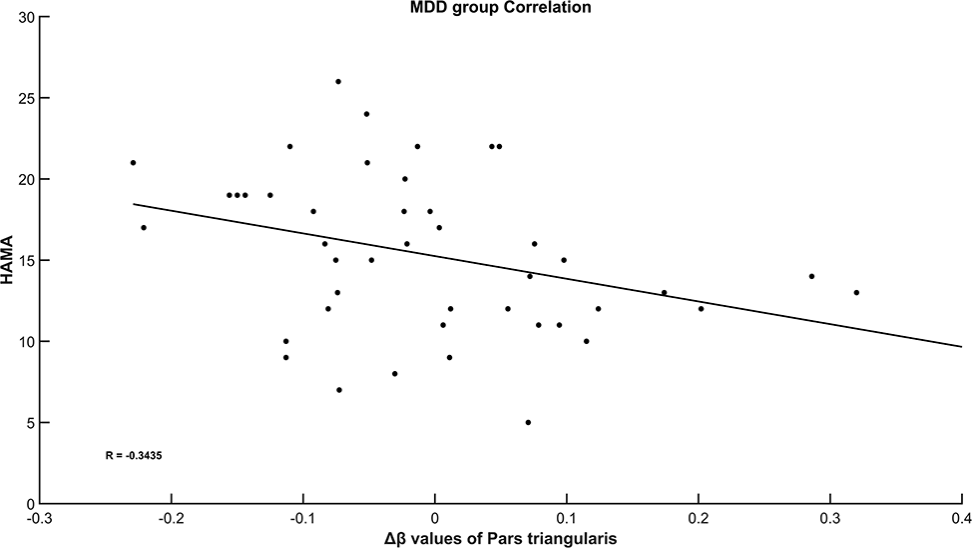
The correlation of Δβ of OFC and HAMA score in the MDD group

The Δβ in all the six brain areas was not correlated with the HAMD score in the minor depression group, see Table 3. Negative correlation was observed between the Δβ in the pars triangularis and the HAMA score in the MDD group, see Table 4; Fig. 3. After an FDR correction, the correlation was not observed.

**Table 3:** The correlation of clinical symptoms and Δβ in the minor depression group

		DLPFC	Frontopolar area	Subcentral area	OFC	MTG	pars triangularis
HAMD	*r*	0.1773	0.2035	0.1155	0.2475	-0.0803	0.1700
	*p*	0.1791	0.1221	0.3838	0.0588	0.5453	0.1981
HAMA	*r*	0.0576	0.0988	0.0780	0.0663	-0.1673	0.0203
	*p*	0.6650	0.4568	0.5568	0.6178	0.2053	0.8785

**Table 4:** The correlation of clinical symptoms and Δβ in the MDD group

		DLPFC	Frontopolar area	Subcentral area	OFC	MTG	pars triangularis
HAMD	*r*	-0.1998	-0.0746	-0.1200	-0.0423	-0.0382	-0.2797
	*p*	0.2104	0.6432	0.4548	0.7927	0.8124	0.0766
HAMA	*r*	-0.1854	-0.1935	-0.1177	-0.1863	-0.1172	-0.3435
	*p*	0.2458	0.2255	0.4638	0.2435	0.4654	0.0279

## Discussion

The current study compared the oxy-hemoglobin response during VFT of the minor depression group, MDD group and HC group. Pervasively lower activation was observed in the MDD group compared to the HC group, however, only the Pars triangularis in the minor depression group exhibited lower activity than the HC group. Neither depressive nor anxious symptoms were correlated with the activation in the minor depression and MDD groups.

In terms of activation of various brain areas in the MDD group, Manelis et al. [31] also reported lower right PFC (prefrontal cortex) activation in the depression group when performing a task looking at recognition of happy facial expressions. Baik et al. [32] found relatively less left oxy-Hb changes in MDD patients compared with HCs during the VFT. The results of Husain et al. [33] were consistent with our study, as they also observed less activation in oxy-hemoglobin in the frontal and temporal cortices in the MDD group. Liu et al. reported significantly less cortical activation in the PFC in adolescents with depression during the 60s task period [25].

For the oxy-hemoglobin brain areas in the minor depression group, Wang et al. divided 41 college students into higher and lower depressive tendencies groups based on BDI score, higher deactivation was observed in the higher depressive tendency group for the following brain areas: the inferior frontal gyrus (BA45), the superior external frontal cortex (BA46), premotor cortex (BA6), and the primary motor cortex (BA4) [24]. This is slightly similar to the current study, as only the pars triangularis (BA45) was observed in our study.

In the present study, no correlation was observed between clinical symptoms and cortical activation in the minor depression group and MDD group after correction for FDR, suggesting that changes of Oxy-Hb may not be related with depressive and anxious symptoms. The results of Tsujii et al. are the same as our study, in that Tsujii et al. also did not find a correlation between the value of the mean change in oxy-Hb during the VFT task and the HAMD value in MDD group [34]. Liu and colleagues reported a negative correlation between the HAMD value and mean Oxy-Hb change values in adolescents with depressive disorder, and no correlation between HAMA scores and mean Oxy-Hb change values across channels, which is partially consistent with ours [25]. Some of the results in previous studies were different from the present study. Uemura et al. divided 80 elderly subjects into depressive and non-depressive groups based on GDS (Geriatric Depression Scale) scores, and used fNIRS to explore the relationship between depressive symptoms and changes in Oxy-Hb in older adults under a cognitive task, showing that the severity of depressive symptoms had a significant negative correlation with the change in Oxy-Hb values in PFC [35]; Liu et al. found that changes in mean Oxy-Hb in patients with Major Depressive Disorder were positively correlated with HAMA and HAMD scores significantly, with the highest correlation with HAMA in bilateral and dorsal–median prefrontal cortex and the highest correlation with HAMD in DLPFC [36]. The potential reasons for the current results may be as follows: First, different cognitive tasks were used in different studies. Second, pathological alterations are a dynamic process and the extent of pathological alterations in various regions of the patient’s brain varies across studies, affecting cerebral hemodynamic responses differently, and as a result, different results may occur. Lastly, the samples are different across different studies, as some studies included patients with MDD, others included healthy participants; the duration of illness may also be a potential factor.

These results suggest that both minor depression and MDD can impair cognitive functioning in adolescents and that minor depression is no less detrimental to adolescent brain function than MDD. Based on our results, it is clear that diagnostic clarity of minor depression should be improved and early intervention should be implemented for patients with minor depression.

In conclusion, the hemodynamic responses of Oxy-Hb of the MDD group were pervasively lower, but only one brain area was lower in the minor depression group.

The cognitive impairment in patients with minor depression cannot be ignored and patients with minor depression have a significantly increased risk of developing into major depressive disorder. Early identification and treatment at the earliest stages of minor depression may change the development of minor depression.

### Limitation

There are several limitations in the current study. First, as the demographic data difference exists in the three groups, however, but for further analysis, the demographic data were covariated. Second, the present study was focused on adolescents only and the sample size was small, future studies should be expanded to other age groups and increase the sample size to generate more generalized results. Lastly, this study is cross-sectional research and we would like to continue to follow-up with the minor depression group for increased understanding.

## Conclusion

The HAMD and HAMA scores were higher in the MDD group and slightly higher in the group with minor depression. The fNIRS showed a slight reduction in cortical activation in the minor depression group and a significant reduction in cortical activation in the MDD group. However, the degree of cortical activation was not related to depressive and anxious scores. These results suggest that reduced cognitive abilities in adolescents with minor depression and MDD, interventions in the early stages of minor depression may help slow or even modify the development of the illness.

**Declarations**.

## Data Availability

Not applicable.

## References

[1] Judd LL, Rapaport MH, Paulus MP (1994). Subsyndromal symptomatic depression: a new mood disorder?. J Clin Psychiatry.

[2] Fournier JC, DeRubeis RJ, Hollon SD, Dimidjian S, Amsterdam JD, Shelton RC (2010). Antidepressant Drug effects and Depression Severity: a patient-level Meta-analysis. JAMA.

[3] Rucci P (2003). Subthreshold psychiatric disorders in primary care: prevalence and associated characteristics. J Affect Disord.

[4] Veerman JL, Dowrick C, Ayuso-Mateos JL, Dunn G, Barendregt JJ (2009). Population prevalence of depression and mean Beck Depression Inventory score. Br J Psychiatry.

[5] Rowe SK, Rapaport MH (2006). Classification and treatment of sub-threshold depression. Curr Opin Psychiatry.

[6] Tuithof M, ten Have M, van Dorsselaer S, Kleinjan M, Beekman A, de Graaf R (2018). Course of subthreshold depression into a depressive disorder and its risk factors. J Affect Disord.

[7] Thapar A, Eyre O, Patel V, Brent D (2022). Depression in young people. Lancet.

[8] Ghazi Sherbaf F, Same K, Ashraf-Ganjouei A, Aarabi MH (2018). Altered white matter microstructure associated with mild and moderate depressive symptoms in young adults, a diffusion tensor imaging study. NeuroReport.

[9] Polyakova M, Mueller K, Sander C, Beyer F, Witte V, Lampe L (2018). No changes in Gray Matter density or cortical thickness in late-life minor depression. J Clin Psychiatry.

[10] Javaheripour N, Colic L, Opel N, Li M, Maleki Balajoo S, Chand T (2023). Altered brain dynamic in major depressive disorder: state and trait features. Transl Psychiatry.

[11] Li J, Wang R, Mao N, Huang M, Qiu S, Wang J (2023). Multimodal and multiscale evidence for network-based cortical thinning in major depressive disorder. NeuroImage.

[12] Zhang J, Wang J, Wu Q, Kuang W, Huang X, He Y (2011). Disrupted brain connectivity networks in Drug-Naive, first-episode major depressive disorder. Biol Psychiatry.

[13] Zhang Z, Zhang H, Xie C-M, Zhang M, Shi Y, Song R (2021). Task-related functional magnetic resonance imaging-based neuronavigation for the treatment of depression by individualized repetitive transcranial magnetic stimulation of the visual cortex. Sci China Life Sci.

[14] Ma Z, Li R, Yu J, He Y, Li J (2013). Alterations in Regional Homogeneity of spontaneous brain activity in late-life Subthreshold Depression. PLoS ONE.

[15] Ho CSH, Lim LJH, Lim AQ, Chan NHC, Tan RS, Lee SH (2020). Diagnostic and predictive applications of Functional Near-Infrared spectroscopy for major depressive disorder: a systematic review. Front Psychiatry.

[16] Yeung MK, Lin J (2021). Probing depression, schizophrenia, and other psychiatric disorders using fNIRS and the verbal fluency test: a systematic review and meta-analysis. J Psychiatr Res.

[17] Ferrari M, Quaresima V (2012). A brief review on the history of human functional near-infrared spectroscopy (fNIRS) development and fields of application. NeuroImage.

[18] Chen W-L, Wagner J, Heugel N, Sugar J, Lee Y-W, Conant L (2020). Functional Near-Infrared Spectroscopy and its clinical application in the field of Neuroscience: advances and future directions. Front Neurosci.

[19] Jalalvandi M, Riyahi Alam N, Sharini H, Hashemi H, Nadimi M (2021). Brain cortical activation during Imagining of the wrist Movement using functional Near-Infrared Spectroscopy (fNIRS). J Biomed Phys Eng.

[20] Jalalvandi M, Sharini H, Naderi Y, RiahiAlam N. Assessment of brain cortical activation in passive movement during wrist task using functional near infrared spectroscopy (fNIRS). Front Biomed Technol. 2019;6:99–105

[21] Sharini H, Fooladi M, Masjoodi S, Jalalvandi M, Yousef Pour M (2019). Identification of the Pain process by Cold Stimulation: using Dynamic Causal modeling of effective connectivity in Functional Near-Infrared Spectroscopy (fNIRS). IRBM.

[22] Dong S-Y, Choi J, Park Y, Baik SY, Jung M, Kim Y (2021). Prefrontal Functional Connectivity during the Verbal Fluency Task in patients with major depressive disorder: a functional Near-Infrared Spectroscopy Study. Front Psychiatry.

[23] Kim H, Choi J, Jeong B, Fava M, Mischoulon D, Park MJ (2022). Impaired oxygenation of the Prefrontal Cortex during Verbal Fluency Task in Young adults with major depressive disorder and suicidality: a functional Near-Infrared Spectroscopy Study. Front Psychiatry.

[24] Wang L, Ke J, Zhang H (2022). A functional Near-Infrared Spectroscopy examination of the neural correlates of Mental Rotation for individuals with different depressive tendencies. Front Hum Neurosci.

[25] Liu X, Cheng F, Hu S, Wang B, Hu C, Zhu Z (2022). Cortical activation and functional connectivity during the verbal fluency task for adolescent-onset depression: a multi-channel NIRS study. J Psychiatr Res.

[26] Sheehan DV. The mini-international neuropsychiatric interview (MINI): the development and validation of a structured diagnostic psychiatric interview for DSM-IV and ICD-10. J Clin Psychiatry.1998;59:22–33. 9881538

[27] Jang KE, Tak S, Jung J, Jang J, Jeong Y, Ye JC (2009). Wavelet minimum description length detrending for near-infrared spectroscopy. J Biomed Opt.

[28] Ye JC, Tak S, Jang KE, Jung J, Jang J (2009). NIRS-SPM: statistical parametric mapping for near-infrared spectroscopy. NeuroImage.

[29] Benjamini Y, Drai D, Elmer G, Kafkafi N, Golani I (2001). Controlling the false discovery rate in behavior genetics research. Behav Brain Res.

[30] Singh AK, Dan I (2006). Exploring the false discovery rate in multichannel NIRS. NeuroImage.

[31] Manelis A, Huppert TJ, Rodgers E, Swartz HA, Phillips ML (2019). The role of the right prefrontal cortex in recognition of facial emotional expressions in depressed individuals: fNIRS study. J Affect Disord.

[32] Baik K, Choi, Baek, Park, Kim (2019). Prefrontal asymmetry during cognitive tasks and its relationship with suicide ideation in major depressive disorder: an fNIRS Study. Diagnostics.

[33] Husain SF, Yu R, Tang T-B, Tam WW, Tran B, Quek TT (2020). Validating a functional near-infrared spectroscopy diagnostic paradigm for major depressive disorder. Sci Rep.

[34] Tsujii N, Mikawa W, Akashi H, Tsujimoto E, Adachi T, Kirime E (2014). Right temporal activation differs between melancholia and nonmelancholic depression: a multichannel near-infrared spectroscopy study. J Psychiatr Res.

[35] Uemura K, Shimada H, Doi T, Makizako H, Park H, Suzuki T (2014). Depressive symptoms in older adults are associated with decreased cerebral oxygenation of the prefrontal cortex during a trail-making test. Arch Gerontol Geriatr.

[36] Liu X, Sun G, Zhang X, Xu B, Shen C, Shi L (2014). Relationship between the prefrontal function and the severity of the emotional symptoms during a verbal fluency task in patients with major depressive disorder: a multi-channel NIRS study. Prog Neuropsychopharmacol Biol Psychiatry.

